# WAD-YOLO: A Lightweight Fall Detection Algorithm for Visual Sensor Systems Based on Wavelet Transform and Dynamic Convolution

**DOI:** 10.3390/s26134199

**Published:** 2026-07-02

**Authors:** Zhongyu He, Fenghua Zhu, Shengli Duan, Xiaowei Li, Zhenyu Shen, Yuanlin Wang

**Affiliations:** 1 School of Rail Transportation, Shandong Jiaotong University, Jinan 250300, China; 24221007@stu.sdjtu.edu.cn (Z.H.); 215025@sdjtu.edu.cn (S.D.); 24221016@stu.sdjtu.edu.cn (Z.S.); 24221023@stu.sdjtu.edu.cn (Y.W.); 2 Beijing Huairou Parallel Sensing Intelligence Research Institute, Beijing 101400, China; fenghua.zhu@ia.ac.cn

**Keywords:** fall detection, visual sensor system, wavelet transform, dynamic convolution, lightweight network, edge deployment, YOLOv11

## Abstract

Falls among the elderly and vulnerable populations represent a critical public health challenge, and camera-based visual sensor systems have emerged as a promising non-intrusive solution for continuous fall monitoring. However, deploying accurate fall detection on resource-constrained edge sensor nodes remains difficult due to the trade-off between model complexity and detection performance. In this paper, we propose WAD-YOLO, an efficient and lightweight fall detection algorithm tailored for visual sensor systems, based on wavelet transform and dynamic convolution. First, a wavelet transform convolution (WTConv) module is introduced to expand the receptive field of the visual feature extractor via cascaded wavelet decomposition, enabling the sensor-driven model to better capture low-frequency fall-related patterns without parameter explosion. Second, a dynamic upsample (DySample) operator is incorporated into the detection head to achieve content-aware, flexible upsampling by generating dynamic offsets, maintaining high efficiency suitable for real-time sensor data processing. Third, an adaptive downsampling (ADown) module is integrated to reduce spatial resolution while preserving semantic information, further reducing the computational burden for deployment on embedded sensor platforms. Experiments on the public Fall Detection dataset demonstrate that, compared with the baseline YOLOv11n, the proposed method increases precision P by 3.8%, mAP50 by 3.7%, and reduces the parameter count by 3.0 × 10^5^. The reduced parameter count and matched GFLOPs relative to YOLOv11n suggest that WAD-YOLO is a theoretically promising candidate for lightweight, high-accuracy fall detection on edge sensor platforms.

## 1. Introduction

With the intensification of global population aging, falls have become a major threat to physical health. According to data from the World Health Organization, approximately 37.3 million falls require medical intervention each year, with about 600,000 cases leading to death. Timely detection can reduce the risk of death by 80%. Therefore, it has become crucial to study pedestrian fall detection technology based on intelligent sensing systems.

Among the various sensing modalities available for fall detection, camera-based visual sensor systems have attracted significant attention due to their non-intrusive nature, rich spatial information, and wide deployment in smart home, hospital, and public surveillance environments. Unlike wearable sensors that require user compliance, visual sensors can operate continuously and passively, making them well-suited for long-term monitoring of elderly individuals. However, deploying deep learning-based fall detection algorithms on resource-constrained edge sensor nodes—such as embedded cameras, IoT gateways, and smart surveillance devices—poses a fundamental challenge: achieving high detection accuracy while maintaining a lightweight model footprint compatible with limited computational and memory resources.

In the field of fall detection, the current technical methods mainly include the following three categories: methods based on human feature detection, methods based on scene intelligent perception, and methods based on visual recognition.

Human feature detection methods have detection effects on specific datasets, but some need to optimize generalization or practical application performance. Luo et al. [1] proposed a real-time fall detection method that combines FMCW radar and LACNN network. By using radar to extract micro-motion features and enhancing the feature representation ability through a hierarchical attention convolutional neural network, a high detection accuracy was achieved on the self-built radar dataset with a delay of less than 50 ms. However, this dataset cannot cover the complex and changing fall actions in life, and the generalization ability needs to be strengthened. Xiao et al. [2] integrated STRS-YOLO with OpenPose pose estimation and analyzed human action sequences through the spatio-temporal feature fusion module, effectively improving the recognition accuracy and robustness on the FDD dataset. Wan et al. [3] processed sensor time series data based on ResNet18 and compared it with traditional machine learning methods on public sensor datasets. Optimization is needed for specific situations to enhance accuracy and reliability in practical applications.

Scene intelligent perception methods have a relatively high accuracy rate in fall detection in different scenarios, but some methods rely on specific hardware or large-scale dedicated datasets. Antonello et al. [4] utilized the 3D depth sensor of mobile robots, combined with hypersegmentation and an SVM classifier for spatial relationship analysis, achieving an accuracy rate of 92% in detecting fallen personnel in complex scenes, and integrated robot navigation and map verification functions. Cheng et al. [5] conducted experiments with Raspberry Pi as the core, integrating cameras, six-axis sensors, positioning modules, heart rate modules and NB-IoT modules. The results showed that the accuracy rates of fall detection in outdoor and indoor environments reached 92.8% and 91.0% respectively. Khan et al. [6] optimized YOLOv8 by integrating the Focus module and the convolutional block attention module CBAM, and trained it on a diverse large-scale fall detection dataset (DiverseFALL10500), significantly enhancing the model’s adaptability in complex scenarios.

Computer vision recognition methods are currently quite mature. Xun et al. [7] proposed the PCE-YOLO model, integrating the CPA-Enhancer module and the Inner EIoU loss function. In scenarios such as low light and occlusion, mAP50-95 was 4.52% higher than YOLOv8n, and the FPS reached 210.5. Wang et al. [8] designed the LFD-YOLO model, integrating the CSRG lightweight module with the WFPN feature pyramid, and combining the Inner-WIoU loss function. On the PFDD dataset, mAP50 improved by 1.5% compared to YOLOv5, and the parameters were reduced by 19.2%. Huang et al. [9] introduced the SDFP multi-scale pyramid and SEAM occluded attention, combined with the WIoU-Shape loss function to design the SDES-YOLO model. On the publicly available Fall Detection dataset, the mAP50 reached 85.1%, and the parameter count was only 2.9 M. Ren et al. [10] integrated CSPNet and ELAN into the GELAN module, enhancing accuracy while maintaining the efficiency of YOLOv8. Nagaj et al. [11] utilized SAM to separate the foreground from the background and applied Gaussian blur to the background to suppress context interference. On the merged datasets such as CAUCAFall, the F1 Score of EfficientNetV2-S increased by 4.2%. Liu et al. [12] proposed HFDMIA-Pose, fusing the SPD-ConV-optimized YOLOv8s with AlphaPose. On self-built multi-person scene datasets such as MPFDD, compared with the original AlphaPose, the average accuracy was increased by 4.3%, F1 was increased by 4.57%, and FPS was increased by 37.5%. Ye et al. [13] proposed the RRES-YOLO model. On the self-built home fall dataset, the mAP50 was improved by 2.1%, the number of parameters was reduced by 19.4%, and the computational load was decreased by 9.2%, demonstrating higher robustness in environments with varying lighting, occlusion, and complex home settings involving multiple people. Feng et al. [14] introduced the convolutional attention mechanism (CBAM) and the weighted bidirectional feature pyramid network (BiFPN) on the basis of YOLOv5s. The experimental results showed that the model exhibited higher robustness in scenarios with illumination changes, occlusion, and complex multi-person scenarios.

However, there are obvious limitations and deficiencies in the above-mentioned fall detection methods. Cheng et al. [5] rely on hardware such as wearable devices. Although they can achieve fall detection to a certain extent, such detection schemes based on wearable devices have problems such as poor wearing compliance and high cost. Zhang et al. [15] extracted the detection target by improving the Vibe algorithm and then used the support vector machine (SVM) to distinguish between falls and other states. However, this method has certain false detections for lying down and quickly sitting down states.

The development of deep learning technology provides new ideas for solving the above problems, but existing models still face challenges when integrated into real-world visual sensor systems: The target detection accuracy is relatively low, the adaptability to complex scenes with multiple human bodies is poor, and the model lightweighting during deployment on edge sensor hardware is insufficient [16]. Among them, the representative algorithms include SSD [17], Fast R-CNN [18], Faster R-CNN [19], RetinaNet [20], etc. For instance, the YOLO series [21] has limited capabilities in extracting target features in complex scenarios such as low light and occlusion. The LFD-YOLO [8] model still faces challenges in addressing complex background interference and distinguishing similar behaviors. Although SDES-YOLO [9] emphasizes its applicability in various practical fields, it still needs to further reduce computational requirements and increase processing speed to meet deployment needs.

It is also worth noting that non-vision-based fall detection methods, such as WiFi channel state information (CSI)-based approaches, have recently gained attention as privacy-preserving alternatives to camera-based systems. Boudlal et al. [22] proposed a low-cost indoor activity recognition system using WiFi CSI, demonstrating that human activities can be recognized without cameras by analyzing wireless signal fluctuations. Similarly, Boudlal et al. [23] developed an optimized GRU-RNN framework for real-time human interaction recognition using WiFi CSI, further highlighting the potential of non-visual modalities for activity monitoring. While these approaches offer important privacy advantages, they generally exhibit lower spatial resolution and limited ability to distinguish fine-grained postures (e.g., distinguishing a fall from quickly sitting down), motivating the continued development of lightweight visual methods that can be deployed with appropriate privacy safeguards.

In view of the above challenges, this paper presents the following technical innovations, and proposes WAD-YOLO, an efficient and lightweight fall detection model based on wavelet transform and dynamic convolution, specifically designed for deployment on visual sensor systems:

**Optimize detection accuracy:** Utilize wavelet transform to expand the receptive field of convolution, and through cascade wavelet decomposition, enable the CNN to better respond to low frequencies, achieving efficient capture of multi-scale information from visual sensor inputs.

**Scene adaptability enhancement:** By generating dynamic offsets to adjust the sampling position and combining bilinear interpolation and pixel shuffling techniques, flexible and efficient upsampling operations are achieved, improving robustness across diverse sensor-captured scenes including low-light, occlusion, and motion blur.

**Lightweight design for edge sensor deployment:** Introduce an adaptive downsampling module [24] to reduce the spatial dimension of the feature map and lower the spatial resolution, while maintaining the model’s lightweight and detection accuracy. The resulting low parameter count and computational footprint indicate that the model is, in principle, well-matched to resource-constrained embedded visual sensor platforms such as IoT edge nodes and smart surveillance cameras.

**Statement of Novelty:** Compared with existing fall detection methods, the novelty of WAD-YOLO lies in three aspects. First, this is the first work that integrates cascaded wavelet transform convolution (WTConv) into the YOLO detection framework specifically for fall detection, enabling frequency-domain receptive field expansion without parameter explosion. Second, we propose a novel combination of point-sampling-based dynamic upsampling (DySample) with adaptive downsampling (ADown) in the detection head, achieving content-aware feature refinement while significantly reducing model complexity. Third, the synergistic design of these three modules achieves simultaneous improvements in both detection accuracy (+3.7% mAP50) and model compactness (−3.0 × 10^5^ parameters) over the YOLOv11n baseline, which is a non-trivial outcome rarely achieved in prior lightweight detection methods.

## 2. WAD-YOLO Model

**Figure 1:**
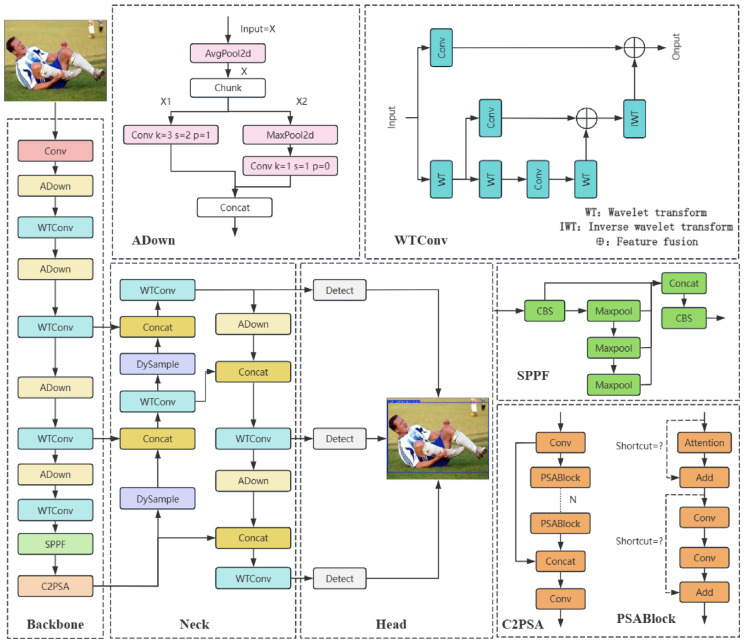
The network structure of the WAD-YOLO model.

The network structure of YOLOv11 [25] can be divided into three main parts: the backbone, the neck and the head network. As illustrated in Figure 1, the proposed WAD-YOLO integrates three key modules into the YOLOv11n baseline at specific positions:

**(1) WTConv in the Backbone and the Neck:** Wavelet transform convolution (WTConv) optimizes the standard convolution within the C3k2 modules in the backbone network. The core idea is to leverage wavelet transform to achieve efficient multi-scale feature extraction while significantly expanding the receptive field without parameter explosion.

**(2) ADown in the Backbone and the Neck:** The adaptive downsampling module (ADown) optimizes the conventional strided convolution blocks used for spatial downsampling in the neck network. ADown reduces the spatial dimension of the feature map through a combination of average pooling and split convolutions, preserving more semantic information while reducing computational cost.

**(3) DySample in the Neck:** The dynamic upsample operator (DySample) optimizes the nearest-neighbor upsampling operations in the detection head. By dynamically adjusting the positions of sampling points and combining bilinear interpolation with pixel shuffling [26] techniques, DySample achieves content-aware, flexible upsampling with minimal overhead.

The multi-scale feature fusion in the neck is accomplished through Concat operations, which concatenate features from different backbone levels with the corresponding upsampled or downsampled features from adjacent levels, forming the feature pyramid for detection at three scales.

### 2.1. Wavelet Convolution

In the field of object detection, convolutional neural network (CNN) is a mainstream feature extraction tool [27]. However, traditional convolution encounters problems such as excessive parameters and high computational costs when dealing with large receptive fields. Wavelet transform convolution (WTConv) utilizes wavelet transform (WT) to significantly expand the receptive field of convolution and guides the CNN to better respond to low frequencies through cascading wavelet decomposition. This technology effectively increases the receptive field of convolution by using signal processing tools [28] without being affected by excessive parameterization.

Wavelet transform is a powerful signal processing and analysis tool that can decompose signals into sub-bands of different frequencies. In the field of images, two-dimensional wavelet transforms (such as Haar WT) can decompose images into four subbands: the low-frequency subband (LL) mainly captures the global structural information of the image; the horizontal high-frequency subband (LH), vertical high-frequency subband (HL), and diagonal high-frequency subband (HH) respectively capture the detailed information of the image in the horizontal, vertical, and diagonal directions [29]. WTConv effectively combines wavelet transform with convolution operations. First, perform wavelet transform decomposition on the input image or feature map. The initial input serves as the lowest-level low-frequency component, and then through cascade wavelet decomposition, each level further decomposes the low-frequency component of the previous level into four subbands. After each level of decomposition, small kernel convolution (such as 3 × 3 or 5 × 5 convolution kernels) operations are respectively applied to each subband. Small-kernel convolution is carried out independently on different frequency bands without interfering with each other, thus effectively avoiding the problem of over-parameterization.

Given an image X, for each input channel, the convolution output has four channels, and the resolution of each channel (in each spatial dimension) is half that of image.

(1) $$\left[X_{LL},X_{LH},X_{HL},X_{HH}\right]=Conv\left(\left[f_{LL},f_{LH},f_{HL},f_{HH}\right],X\right)$$

In the formula: $X_{LL}$ is the low-frequency component of X, while $X_{LH}$, $X_{HL}$ and $X_{HH}$ are the horizontal, vertical and diagonal high-frequency components of X respectively; $f_{LL}$ is a low-pass filter, while $f_{LH}$, $f_{HL}$ and $f_{HH}$ are a group of high-pass filters.

Since the kernels in Equation (1) form orthogonal bases, the inverse wavelet transform (IWT) can be obtained through transposition convolution. Then, the cascaded wavelet decomposition is given by recursively decomposing the low-frequency components. Each level of decomposition is given by the following formula:

(2) $$X_{LL}^{\left(i\right)},X_{LH}^{\left(i\right)},X_{HL}^{\left(i\right)},X_{HH}^{\left(i\right)}=WT\left(X_{LL}^{\left(i-1\right)}\right)$$

Here $X_{LL}^{\left(0\right)}=X$, *i* represents the current level. This will increase the frequency resolution while reducing the spatial resolution at lower frequencies.

Increasing the kernel size in traditional convolutional layers will lead to a quadratic growth in the number of parameters. To solve this problem, first use WT to filter and reduce the low-frequency and high-frequency content of the input. Then, perform small-kernel depth convolution on different frequency maps and construct the output using IWT. This process is given by the following formula:

(3) $$Y=\mathrm{IWT}\left(Conv\left(W,\mathrm{WT}\left(X\right)\right)\right)$$

**Figure 2:**
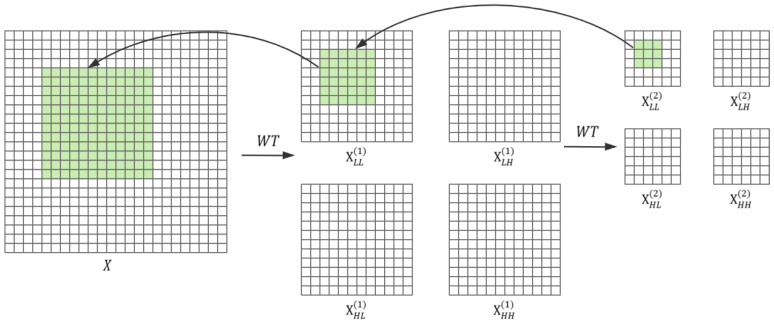
Performing convolution in the wavelet domain generates a larger receptive field. The green area in *X* represents the input region for the original low-frequency features; the green area within $X_{LL}^{\left(1\right)}$ is the low-frequency subband area after the first-level wavelet decomposition; the green area within $X_{LL}^{\left(2\right)}$ is the low-frequency subband area after the second-level wavelet decomposition.

Here, X is the input tensor, and W is the k × k depth convolution kernel weight tensor. This operation enables the smaller kernel to run within a larger area of the original input, achieving the effect of increasing the receptive field. As shown in Figure 2.

Further enhance it using the same cascading principle as in Equation (2). This process is given by the following formula:

(4) $$X_{LL}^{\left(i\right)},X_{H}^{\left(i\right)}=WT\left(X_{LL}^{\left(i-1\right)}\right)$$

(5) $$Y_{LL}^{\left(i\right)},Y_{H}^{\left(i\right)}=Conv\left(W^{\left(i\right)},\left(X_{LL}^{\left(i\right)},X_{H}^{\left(i\right)}\right)\right)$$

Among them, $X_{H}^{\left(i\right)}$ represents all three high-frequency graphs of level *i*; $Y_{LL}^{\left(i\right)}$ represents the low-frequency output feature after convolution operation on the low-frequency subband obtained by wavelet decomposition at the *i*-th level. $Y_{H}^{\left(i\right)}$ represents the high-frequency output feature after convolution operation on all high-frequency subbands obtained after wavelet decomposition at the *i*-th level.

To combine the outputs of different frequencies, the linear properties of the wavelet transform and its inverse transform are utilized to merge the outputs of different frequencies. The result is the sum of convolution at different levels. This process is given by the following formula:

(6) $$Z^{\left(i\right)}=IWT\left(Y_{LL}^{\left(i\right)}+Z^{\left(i+1\right)},Y_{H}^{\left(i\right)}\right)$$

Among them, $Z^{\left(i\right)}$ represents the final multi-scale fusion feature map obtained.

### 2.2. Lightweight Dynamic Upsampling Operator

The existing upsampling methods based on dynamic convolution (such as CARAFE, FADE, etc.) have problems such as large computational load and reliance on high-resolution guided features. DySample, as an ultra-lightweight dynamic upsampling operator, redefines the upsampling process from the perspective of point sampling. It bypasses the heavy workload brought by dynamic convolution, returns to the essence of upsampling, and redefines the upsampling process, that is, generates content-aware sampling points to re-sample the continuous mapping. In summary, DySample adjusts the sampling position by generating dynamic offsets and combines highly optimized built-in functions in PyTorch to achieve lightweight and efficient upsampling operations.

**Figure 3:**
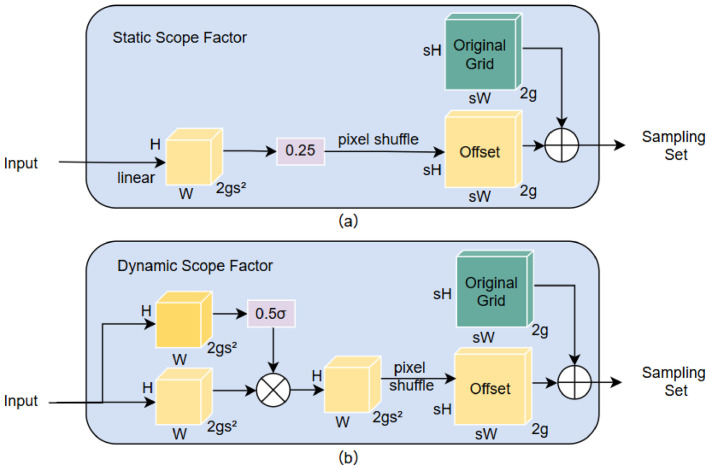
The sampling point generator in DySample. (**a**) Static Scope Factor Branch; (**b**) Dynamic scope factor branch.

In the implementation of the basic sampling framework, the initial offset positions shared among sampling points on $s^{2}$ ignore the positional relationship, and the unconstrained movement range of the offset will lead to the disorder of point sampling. For these two problems, this method changes the initial position to “bilinear initialization”. However, due to the existence of the normalization layer, the value of a certain output feature is usually within the range of [−1, 1] centered on 0. Therefore, the moving range of the local $s^{2}$ sampling position may overlap. To alleviate this situation, the offset is multiplied by a static range coefficient of 1/(2s). For an upsampling scale factor of s=2, this coefficient equals 0.25, which is theoretically derived to ensure that the sampling ranges of adjacent output points exactly meet at their boundaries without overlap or gap. This coefficient constrains the walking range of each sampling position locally. As shown in Figure 3a.

To enhance the flexibility of offset, linear projection input features are adopted to further generate point-by-point “dynamic range factors”. The dynamic range applies a sigmoid function (output range [0, 1]) scaled by a 0.5 factor, producing values within the [0, 0.5] range centered on the static coefficient 0.25. This design allows the network to adaptively adjust the sampling range around the theoretically optimal static value while maintaining stable initialization. The dynamic range operation is shown in Figure 3b, and the process is given by the following formula:

(7) $$DS=0.5\cdot sigmoid\left(linear_{1}\left(x\right)\right)\cdot linear_{2}\left(x\right)$$

Next, the channel number $2gs^{2}$ is reshaped into a spatial dimension with a size of sH×sW and a channel number of 2g to obtain the Offset, which is used to record the offset of x and y at each position. Finally, add the basic network lattice and the offset to obtain the final Sampling network Sampling Set.

### 2.3. Adaptive Downsampling Module

In conventional deep learning architectures, spatial downsampling is typically achieved through strided convolution or max pooling, which may discard important semantic information during feature compression. The adaptive downsampling module (ADown), originally proposed in YOLOv9 [24], addresses this issue through a more information-preserving downsampling strategy.

Specifically, ADown first applies average pooling with a kernel size of 2 × 2 to the input feature map, reducing the spatial resolution by half while preserving global context. The pooled features are then split along the channel dimension into two equal halves. One half is processed by a 1 × 1 pointwise convolution for channel-wise feature refinement, while the other half is processed by a 3 × 3 depthwise separable convolution for spatial feature extraction. The two processed branches are concatenated along the channel dimension, followed by a final 1 × 1 convolution for channel adjustment. This split-process-merge design ensures that both fine-grained spatial details and high-level semantic information are retained during downsampling.

From an information-theoretic perspective, this design mitigates the information bottleneck problem [24]. In deep neural networks, information loss during the feedforward process can be described by the inequality:

(8) $$MI\left(X,X\right)\geq MI\left(X,f_{\theta }\left(X\right)\right)\geq MI\left(X,g_{\phi }\left(f_{\theta }\left(X\right)\right)\right)$$

where MI denotes mutual information, $f_{\theta }$ and $g_{\phi }$ represent successive network transformation layers. As network depth increases, the mutual information between the input *X* and the intermediate representations progressively decreases, leading to unreliable gradients. By employing the parallel branch structure with average pooling (which approximates a low-pass filter and preserves low-frequency information), ADown retains more mutual information with the input compared to conventional strided convolution, yielding more reliable gradient estimates for model optimization.

In WAD-YOLO, ADown replaces the standard downsampling convolutions in the neck network (see Figure 1), achieving a reduction of approximately 0.5 M parameters and 1.2 GFLOPs compared to the baseline, while maintaining or improving detection accuracy.

## 3. Experiment and Results

### 3.1. Experimental Environment and Dataset

The experimental environment is as follows: the operating system is 64-bit Windows 11 (Microsoft Corporation, Redmond, WA, USA); the CPU is the 13th Gen Intel i9-13900K (Intel Corporation, Santa Clara, CA, USA); the GPU is NVIDIA GeForce RTX 4090 (NVIDIA Corporation, Santa Clara, CA, USA); the software environment is Python 3.10.18, PyTorch 2.5.1, and CUDA 12.4 (NVIDIA Corporation, Santa Clara, CA, USA).

Relevant parameter configuration: The input image resolution is uniformly set to 640 × 640. The training batch size is set to 16, and the number of training epochs is 100. The SGD optimizer is used with weight decay of 0.0005. The initial learning rate is set to 0.01 with cosine annealing scheduling, gradually decreasing to a final learning rate of 0.0001. Data augmentation techniques include random horizontal flipping, mosaic augmentation, random scaling (±50%), and HSV color jittering (H ± 0.015, S ± 0.7, V ± 0.4). No early stopping is employed; all models are trained for the full 100 epochs and the best-performing checkpoint on the validation set is selected for evaluation. To ensure statistical reliability, all experiments are repeated five times with different random seeds, and results are reported as mean ± standard deviation.

To ensure the applicability of the WAD-YOLO algorithm in different visual sensor deployment scenarios, this paper uses the publicly available Fall Detection dataset [30]. This dataset contains a total of 10,793 images captured by RGB camera sensors, covering various application scenarios such as indoor and outdoor public places, workplaces, sports injuries, and accidental falls, which endows it with extensive practical applicability across different sensor installation environments. The diversity of scenes—including varying illumination conditions, camera angles, and occlusion levels—reflects the real-world challenges faced by visual sensor systems deployed in smart homes, hospitals, and public surveillance infrastructure. The training set contains images of different sizes and has been effectively processed in terms of scale and perspective, such as performing operations like rotation and cropping on the images, as well as processing image attributes (such as gray level and saturation), simulating the variability of sensor-captured data. The original images of this dataset are split into training, validation, and test sets at a ratio of 7:2:1, to guarantee effective support for model training and evaluation.

The key hyperparameters of the three proposed modules are summarized in Table 1.

### 3.2. Evaluation Index

The evaluation metrics selected in this experiment include: Accuracy rate P, which refers to the accuracy rate of the model in detecting the target, reflecting the proportion of actually positive samples among the results predicted by the model as positive samples (i.e., detecting “falls”). The recall rate R represents the proportion of the positive samples correctly predicted by the model among all actual positive samples, reflecting the model’s control ability over missed detections. The Mean Average Precision mAP (Mean Average Precision) represents the mean of AP values in multiple categories, comprehensively reflecting the model’s precision–recall balance ability at different thresholds. The calculation formulas for each indicator are as follows:

(9) $$P=\frac{TP}{TP+FP}$$

(10) $$R=\frac{TP}{TP+FN}$$

(11) $$AP=\int_{0}^{1}p\left(r\right)dr$$

(12) $$mAP=\frac{1}{n}\sum_{i=1}^{n}AP_{i}$$

In the formula: TP (True Positive) represents the samples that are correctly predicted as positive examples; FP (False Positive) is the sample where the false prediction is a positive example; FN (False Negative) represents samples that are actually positive examples but are predicted to be negative examples; AP is the area under the P-R curve of a certain category; n represents the number of detection categories; In the evaluation index mAP, mAP50 represents the mAP when the intersection and union ratio (IoU) threshold is 0.5. mAP50-95 refers to the calculation of mAP at 10 different thresholds ranging from 0.5 to 0.95 with a 0.05 step size for the IoU threshold, and then taking the average.

### 3.3. Comparative Experiment

The proposed WAD-YOLO was compared with mainstream object detection algorithms, including both general-purpose detectors and recently proposed fall-detection-specific models. To ensure fair comparison, all models were trained and evaluated under identical experimental conditions described above. Each experiment was repeated five times, and results are reported as mean ± standard deviation. The results are shown in Table 2.

As shown in Table 2, significant differences exist among the models in terms of detection performance, parameter count, and computational load. Among the general-purpose detectors, YOLOv8 achieves a P of 80.5 ± 0.8%, R of 76.0 ± 1.1%, and mAP50 of 82.3 ± 0.6% with 3.00 M parameters and 8.1 GFLOPs. YOLOv11n serves as our baseline with an mAP50 of 84.6 ± 0.5% and a more compact architecture (2.58 M, 6.3 GFLOPs). Faster R-CNN and RT-DETR-R18 represent two-stage and Transformer-based architectures respectively; while Faster R-CNN achieves a high mAP50 of 86.6 ± 0.4%, its enormous parameter count (41.30 M) and computational cost (113.92 GFLOPs) make it entirely unsuitable for edge deployment.

Among the improved YOLO-based fall detection models, PCE-YOLO achieves a P of 82.5 ± 0.7% and an mAP50 of 84.2 ± 0.5% by integrating the CPA-Enhancer module. However, its parameter count increases to 3.3 M and GFLOPs rise to 9.0. LFD-YOLO achieves a competitive parameter count of 2.5 M, yet its mAP50 of 83.8 ± 0.6% remains moderate. Although SDES-YOLO achieves a P of 84.3 ± 0.6%, its Recall of only 76.0 ± 1.2% limits its practical utility in safety-critical fall detection.

Overall, WAD-YOLO achieves the best overall performance with P of 84.5 ± 0.6%, R of 81.8 ± 0.9%, and mAP50 of 88.3 ± 0.4%. Compared with PCE-YOLO, WAD-YOLO improves P by 2.0%, R by 3.8%, and mAP50 by 4.1%, while reducing parameters by 30.9% and GFLOPs by 30.0%. Compared with LFD-YOLO, WAD-YOLO achieves improvements of 2.5% in P, 5.3% in R, and 4.5% in mAP50, with 8.8% fewer parameters and 10.0% lower GFLOPs. These results confirm that WAD-YOLO achieves a superior balance between detection performance and computational efficiency.

In terms of model lightweighting and efficiency, the parameter count of WAD-YOLO (2.28 M) is 11.6% lower than YOLOv11n (2.58 M) while matching its GFLOPs at 6.3. The parameter counts of Faster R-CNN and RT-DETR-R18 are 41.30 M and 20.18 M respectively, making them impractical for edge deployment. In summary, WAD-YOLO achieves the optimal trade-off between accuracy and efficiency among all compared models.

**Figure 4:**
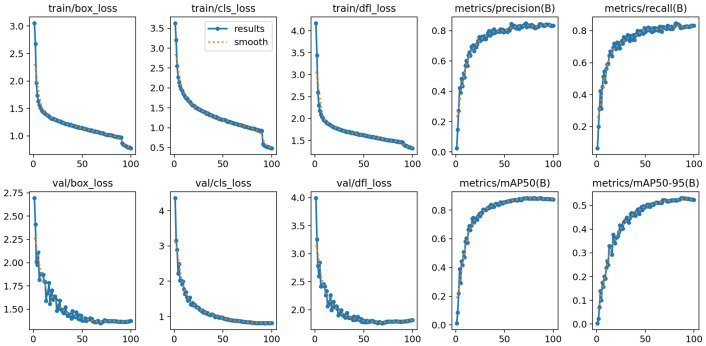
Visualization chart of the training loss and performance index curve of the WAD-YOLO model.

Figure 4 shows the training and validation loss curves alongside the performance index curves of WAD-YOLO. All three loss components (box_loss, cls_loss, dfl_loss) converge smoothly without divergence between training and validation losses, confirming stable optimization and the absence of overfitting. The Precision and Recall both stabilize above 0.8, while mAP50 approaches 0.88 and mAP50-95 reaches approximately 0.53, demonstrating consistent and reliable detection performance across varying IoU thresholds.

The YOLOv11 algorithm has five scales: n, s, m, l, and x. As the number of parameters and the amount of floating-point operations increase successively, the detection performance also improves accordingly. The WAD-YOLO was compared with the YOLOv11 algorithms of various scales, and the results are shown in Table 3. In terms of detection accuracy, the mAP50 of YOLOv11m reaches 88.9%, demonstrating outstanding detection precision and generalization. The Precision of YOLOv11x is 85.0%, performing the best in terms of accuracy indicators. The various indicators of the WAD-YOLO are only slightly inferior to those of YOLOv11m and YOLOv11x.

However, in terms of model complexity and computational efficiency, the parameter count of the WAD-YOLO is only 2.28 × 10^6^ and its GFLOPs is 6.3, which is comparable to that of YOLOv11n. Compared with the YOLO model from s to x versions, where the number of parameters and GFLOPs have been increasing rapidly, the algorithm proposed in this paper achieves high detection accuracy and generalization ability while maintaining lightweight. These complexity-based metrics indicate that WAD-YOLO is, in principle, better matched to resource-constrained scenarios than its larger counterparts; this remains a theoretically motivated expectation, as the corresponding on-device inference performance has not yet been measured in this study.

### 3.4. Analysis of Experimental Results

**Figure 5:**
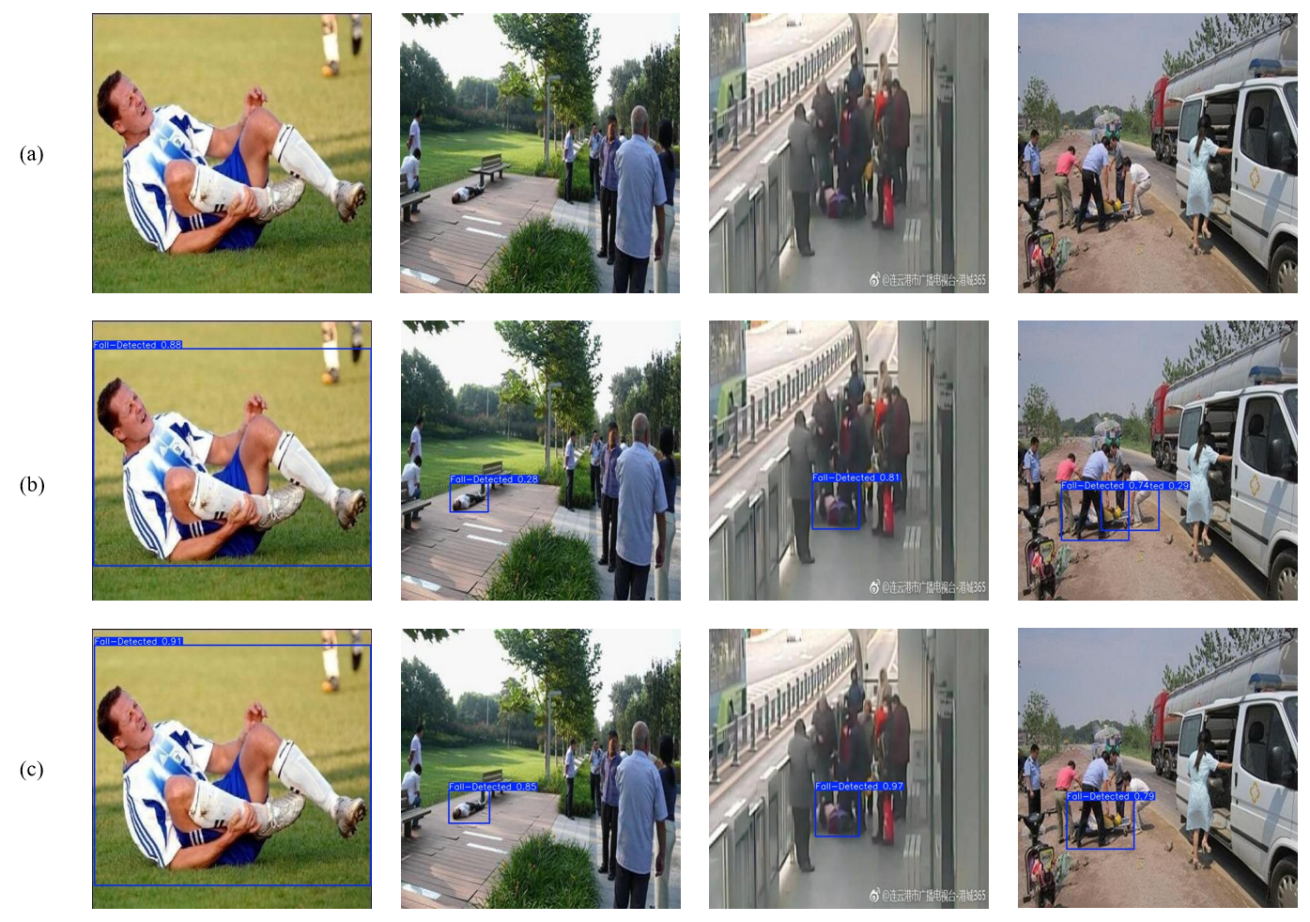
Comparison of model detection results. (**a**) Ordinary images, (**b**) YOLOv11n detection results, (**c**) WAD-YOLO detection results. From left to right are ordinary scene, small target scene, blurry scene quality, and target occlusion scene.

The comparison of model detection effects is shown in Figure 5. In Figure 5, From top to bottom are the original image, the detection result of the YOLOv11n benchmark experiment, and the detection result of the improved model.

The results show that in ordinary scenarios, the WAD-YOLO model has slightly improved the positioning accuracy of falling targets. In small target scenarios, the WAD-YOLO model is more effective for feature extraction and location of small targets, reducing the risks of missed detections and false detections. In scenarios with blurry image quality, although the YOLOv11n model can recognize small targets, its completeness and accuracy are insufficient. The WAD-YOLO model can better capture the human body contours in blurry images, demonstrating its robustness advantage for low-quality images. In the scenario of target occlusion, YOLOv11n is prone to inaccurate box selection and target confusion, while the WAD-YOLO model can clearly identify the target.

In conclusion, the WAD-YOLO model outperforms the benchmark experimental detection results in terms of detection accuracy and robustness in various complex scenarios, effectively enhancing the practicality of the fall detection task.

**Figure 6:**
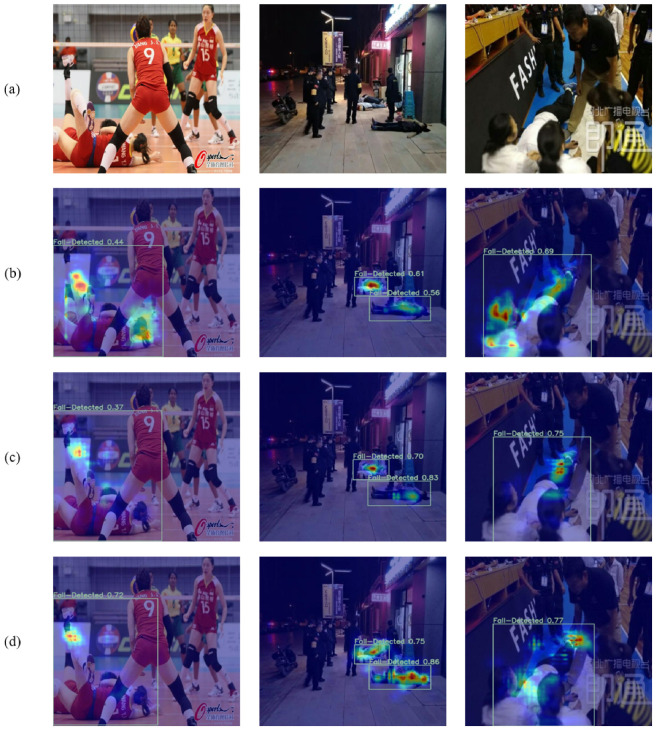
Comparison of model inference heatmap. (**a**) Ordinary images, (**b**) YOLOv11n heatmaps, (**c**) YOLOv8n heatmaps, (**d**) WAD-YOLO heatmaps. From left to right are ordinary scene, multi-objective scene and the scene where the target is blocked. In the heatmaps, red regions indicate high activation areas where the model focuses its attention, while blue regions indicate low activation areas with minimal model response.

The heatmap comparison is shown in Figure 6. In Figure 6, From top to bottom are the original image, the YOLOv11n benchmark experiment, the YOLOv8n comparison model, and the WAD-YOLO model respectively. From the comparison of the model inference heatmap, it can be seen that the WAD-YOLO model, by optimizing feature extraction, significantly reduces the redundant response areas in ordinary, multi-target, and occluded scenarios. This makes the feature extraction of falling targets more accurate and effectively suppresses the false activation of non-falling areas, thereby enhancing the detection confidence and robustness. It outperforms the YOLOv11n benchmark model and the YOLOv8n comparison model.

### 3.5. Ablation Experiment

To evaluate the effects of each module in the WAD-YOLO algorithm, this study selected YOLOv11n as the benchmark model and conducted ablation experiments on the Fall Detection dataset. All seven possible module combinations (three single-module, three pairwise, and one full combination) were evaluated to comprehensively analyze the individual and synergistic effects of each module. Each configuration was trained five times, and results are reported as mean ± standard deviation. The final results are shown in Table 4.

The ablation results in Table 4 reveal both the individual contributions and synergistic interactions of the three modules. Among single-module additions, WTConv yields the largest Precision improvement (+4.4%) through enhanced frequency-domain feature discrimination, while ADown provides the best parameter reduction (from 2.58 M to 2.09 M) with a simultaneous 1.4% mAP50 gain. DySample contributes the most to Recall (+2.7%) via content-aware spatial refinement, with negligible parameter overhead (+0.01 M).

Among pairwise combinations, WTConv + ADown achieves the strongest synergy with mAP50 reaching 87.7%, an improvement of 3.1% over the baseline while reducing parameters to 2.27 M. This synergy arises because WTConv enriches multi-scale frequency features while ADown efficiently compresses spatial dimensions without discarding the enhanced frequency information. In contrast, WTConv+DySample yields a lower mAP50 of 85.2%, suggesting that the frequency enhancement and spatial refinement modules partially overlap in their contributions without ADown’s complementary compression effect.

The full three-module combination achieves the best overall performance (mAP50 = 88.3%), confirming that all three modules contribute non-redundant improvements. The simultaneous gains in both P (+3.8%) and R (+3.6%) over the baseline demonstrate that the synergistic combination successfully mitigates the individual Precision–Recall trade-offs observed with single modules.

The Precision–Recall trade-off across individual modules reflects a fundamental tension in detection system design. WTConv enhances discriminative feature learning through multi-scale frequency decomposition, raising the model’s confidence threshold for positive predictions and thus improving Precision at the cost of slightly reduced Recall. Conversely, DySample improves spatial feature resolution through dynamic upsampling, enabling the model to respond to a broader range of fall-related spatial patterns and thereby increasing Recall, while marginally reducing Precision. In practical fall detection systems, the relative importance of these metrics depends on the deployment context: in clinical or elderly care environments, maximizing Recall should be prioritized, while in public surveillance scenarios, higher Precision may be preferred. The full WAD-YOLO configuration achieves a well-balanced detector suitable for general-purpose fall detection deployment.

### 3.6. Misclassification Analysis

To understand the failure modes of WAD-YOLO, we conducted a detailed analysis of misclassified samples on the test set. The primary error categories are as follows:

**False Positives (FP):** Approximately 62% of false positives are caused by confusion between rapid sitting/lying-down actions and actual falls. These actions share similar spatial configurations (horizontal body orientation, rapid downward motion) and are inherently ambiguous in single-frame detection. Another 15% of false positives arise from individuals bending forward at extreme angles, which produces body poses geometrically similar to early-stage falls.

**False Negatives (FN):** Approximately 23% of false negatives occur under severe occlusion conditions, where the falling person is partially blocked by furniture, other individuals, or environmental obstacles. The remaining false negatives are mainly attributable to extreme distance (very small targets) and unusual fall orientations not well-represented in the training data.

These findings suggest that incorporating temporal information (e.g., video-based sequence modeling) could substantially reduce the confusion between falls and visually similar non-fall actions, as the temporal dynamics of these actions differ significantly. Additionally, training with more diverse occlusion-augmented samples could improve robustness to partial visibility scenarios.

### 3.7. Cross-Dataset Generalization

To evaluate the generalization capability of WAD-YOLO across different data distributions, we conducted an additional experiment on the UR Fall Detection dataset [31]. This dataset, originally collected by the University of Rzeszów, contains 70 video sequences including 30 fall events and 40 activities of daily living (ADLs), captured in a controlled indoor environment using two Microsoft Kinect cameras (RGB + depth). The version used in this study is hosted on Roboflow Universe and provides YOLO-format annotations for the RGB frames. Compared to the primary Fall Detection dataset (10,793 images covering diverse indoor/outdoor scenes), the UR Fall Detection dataset features a single controlled indoor room with a fixed camera viewpoint, representing a distinct data distribution.

Both WAD-YOLO and the baseline YOLOv11n were trained from scratch on the UR Fall Detection training set using the same experimental configuration described in Section 2.1, and evaluated on its test set. Each experiment was repeated five times, and results are reported as mean ± standard deviation. The results are summarized in Table 5.

As shown in Table 5, WAD-YOLO achieves an mAP50 of 94.3% on the UR Fall Detection dataset, outperforming YOLOv11n (91.5%) by 2.8%. The improvement is consistent across all metrics: Precision (+2.4%), Recall (+3.2%), and mAP50-95 (+1.8%). Notably, WAD-YOLO attains a Precision of 97.5% and a Recall of 96.6%, indicating highly reliable detection with minimal false positives and false negatives on this dataset.

The higher overall performance on the UR Fall Detection dataset compared to the primary dataset (mAP50: 94.3% vs. 88.3%) can be attributed to several factors: (1) the UR Fall Detection dataset was captured in a single controlled indoor environment with consistent lighting and a fixed camera angle, resulting in lower visual complexity; (2) the dataset contains clearly distinguishable fall and non-fall activities with less ambiguity compared to the diverse real-world scenes in the primary dataset. Despite these favorable conditions, WAD-YOLO consistently outperforms the baseline across both datasets, confirming the robustness and generalizability of the proposed architectural improvements. The consistent performance advantage of WAD-YOLO over YOLOv11n on both the primary dataset (+3.7% mAP50) and the UR Fall Detection dataset (+2.8% mAP50), with fewer parameters (2.28 M vs. 2.58 M), further validates the effectiveness and transferability of the proposed modules.

## 4. Conclusions and Outlook

This paper proposes WAD-YOLO, an efficient and lightweight fall detection algorithm based on wavelet transform and dynamic convolution, specifically designed for visual sensor systems. By integrating three complementary modules—WTConv for frequency-domain receptive field expansion, DySample for content-aware dynamic upsampling, and ADown for information-preserving adaptive downsampling—WAD-YOLO achieves an mAP50 of 88.3% on the Fall Detection dataset with only 2.28 M parameters and 6.3 GFLOPs, representing a 3.7% improvement over the YOLOv11n baseline while reducing the parameter count by 3.0 × 10^5^. Additional experiments on the UR Fall Detection dataset further validate the generalizability of the proposed method, with WAD-YOLO achieving an mAP50 of 94.3%, outperforming YOLOv11n (91.5%) by 2.8%. It should be emphasized that the low parameter count and matched GFLOPs reported here are theoretical indicators of computational efficiency rather than direct measurements of on-device performance. Accordingly, the suitability of WAD-YOLO for edge sensor deployment is presented in this work as a theoretically motivated expectation rather than an empirically demonstrated outcome; in particular, the wavelet/inverse-wavelet operations of the WTConv module are not standard convolutions, and their behavior under embedded inference engines (e.g., TensorRT/ONNX with FP16/INT8 quantization) remains to be verified through actual on-device benchmarking.

Despite the promising results, several limitations remain. WAD-YOLO operates on single-frame detection without temporal modeling, leading to occasional confusion between falls and visually similar actions (e.g., quickly sitting or lying down). In addition, the current evaluation is limited to RGB imagery and does not cover nighttime or infrared scenarios. Deploying camera-based fall detection also raises privacy concerns, for which non-visual alternatives such as WiFi CSI-based methods [22,23] offer complementary solutions.

Future research will focus on: (1) extending evaluation to more diverse benchmarks and real-world clinical deployments; (2) incorporating temporal modeling to distinguish falls from visually similar activities; (3) exploring multi-modal sensor fusion (e.g., RGB cameras with depth sensors, IMUs, or WiFi CSI); (4) conducting comprehensive inference benchmarking on embedded platforms such as NVIDIA Jetson Nano; and (5) investigating privacy-preserving visual processing pipelines for practical deployment.

## Figures and Tables

**Table 1:** Hyperparameter configuration of the proposed modules.

Module	Parameter	Value	Description
WTConv	Wavelet type	Haar	Orthogonal wavelet basis
	Decomposition levels	2	Cascaded WT depth
	Kernel size	5 × 5	Depthwise conv kernel
DySample	Scale factor *s*	2	Upsampling ratio
	Static range coeff.	0.25	1/(2s)
	Groups *g*	4	Channel groups
ADown	Reduction ratio	0.5	Channel split ratio
	Pooling kernel	2 × 2	Avg pooling size
	Conv kernels	1 × 1, 3 × 3	Branch convolutions

**Table 2:** Comparative experimental data (mean ± std over 5 runs).

Models	P/%	R/%	mAP50/%	Params/M	GFLOPs
YOLOv8	80.5 ± 0.8	76.0 ± 1.1	82.3 ± 0.6	3.00	8.1
YOLOv11	80.7 ± 0.7	78.2 ± 1.0	84.6 ± 0.5	2.58	6.3
Faster R-CNN	46.9 ± 1.5	58.2 ± 1.8	86.6 ± 0.4	41.30	113.92
RT-DETR-R18	77.0 ± 0.9	74.5 ± 1.2	77.8 ± 0.7	20.18	58.6
PCE-YOLO	82.5 ± 0.7	78.0 ± 1.0	84.2 ± 0.5	3.3	9.0
LFD-YOLO	82.0 ± 0.8	76.5 ± 1.1	83.8 ± 0.6	2.5	7.0
SDES-YOLO	84.3 ± 0.6	76.0 ± 1.2	85.1 ± 0.5	2.96	7.2
WAD-YOLO	84.5 ± 0.6	81.8 ± 0.9	88.3 ± 0.4	2.28	6.3

**Table 3:** Comparison experiment with YOLOv11 (mean ± std over 5 runs).

Models	P/%	R/%	mAP50/%	mAP50-95/%	Params/M	GFLOPs
YOLO11n	80.7 ± 0.7	78.2 ± 1.0	84.6 ± 0.5	51.7 ± 0.6	2.58	6.3
YOLO11s	81.6 ± 0.6	84.8 ± 0.8	88.0 ± 0.4	55.1 ± 0.5	9.41	21.3
YOLO11m	84.5 ± 0.5	82.5 ± 0.7	88.9 ± 0.3	57.4 ± 0.4	20.03	67.6
YOLO11l	84.4 ± 0.6	77.9 ± 0.9	85.7 ± 0.5	53.0 ± 0.6	25.28	86.6
YOLO11x	85.0 ± 0.5	83.1 ± 0.7	88.7 ± 0.3	57.1 ± 0.4	56.83	194.4
WAD-YOLO	84.5 ± 0.6	81.8 ± 0.9	88.3 ± 0.4	53.0 ± 0.5	2.28	6.3

**Table 4:** Ablation experimental data (mean ± std over 5 runs). ✓ indicates that the corresponding module is incorporated into the model.

WTConv	DySample	ADown	P/%	R/%	mAP50/%	mAP50-95/%	Params/M	GFLOPs
			80.7 ± 0.7	78.2 ± 1.0	84.6 ± 0.5	51.7 ± 0.6	2.58	6.3
✓			85.1 ± 0.6	76.9 ± 1.1	86.2 ± 0.5	51.4 ± 0.7	2.79	7.5
	✓		80.2 ± 0.8	80.9 ± 0.9	85.9 ± 0.4	51.5 ± 0.6	2.59	6.3
		✓	84.1 ± 0.7	78.0 ± 1.0	86.0 ± 0.5	51.6 ± 0.6	2.09	5.1
✓	✓		81.3 ± 0.8	79.4 ± 1.0	85.2 ± 0.5	50.2 ± 0.7	2.80	7.5
✓		✓	82.8 ± 0.6	81.1 ± 0.8	87.7 ± 0.4	53.8 ± 0.5	2.27	6.3
	✓	✓	80.7 ± 0.7	80.9 ± 0.9	86.9 ± 0.4	52.2 ± 0.6	2.11	5.1
✓	✓	✓	84.5 ± 0.6	81.8 ± 0.9	88.3 ± 0.4	53.0 ± 0.5	2.28	6.3

**Table 5:** Experimental results on the UR Fall Detection dataset (mean ± std over 5 runs).

Model	P/%	R/%	mAP50/%	mAP50-95/%	Params/M	GFLOPs
YOLOv11n	95.1 ± 0.5	93.4 ± 0.6	91.5 ± 0.4	86.8 ± 0.5	2.58	6.3
WAD-YOLO	97.5 ± 0.3	96.6 ± 0.4	94.3 ± 0.3	88.6 ± 0.4	2.28	6.3

## Data Availability

The datasets used in this study are publicly available. The primary Fall Detection dataset is available at https://universe.roboflow.com/roboflow-universe-projects/fall-detection-ca3o8/dataset/4, accessed on 26 May 2025. The UR Fall Detection dataset used for cross-dataset evaluation is available at https://universe.roboflow.com/esanss2/ur-fall-detection-dataset-sz1fh/dataset/1, accessed on 7 June 2026.

## Abbreviations

The following abbreviations are used in this manuscript:

YOLO	You Only Look Once
FMCW	Frequency Modulated Continuous Wave
FDD	Fall Detection Dataset
WTConv	Wavelet Transform Convolution
ADown	Adaptive Downsampling
DySample	Dynamic UpSample
CNN	Convolutional Neural Network
PGI	Programmable Gradient Information
GELAN	Generalized Efficient Layer Aggregation Network
SGD	Stochastic Gradient Descent
CSI	Channel State Information
P	Precision
R	Recall
mAP	Mean Average Precision
GFLOPs	Giga Floating Point Operations Per Second
FPS	Frames Per Second
